# Effects of a Serine Protease Inhibitor N-*p*-Tosyl-L-phenylalanine Chloromethyl Ketone (TPCK) on *Leishmania amazonensis* and *Leishmania infantum*

**DOI:** 10.3390/pharmaceutics14071373

**Published:** 2022-06-29

**Authors:** Patrícia de A. Machado, Pollyanna S. Gomes, Monique P. D. Carneiro, Victor Midlej, Elaine S. Coimbra, Herbert L. de Matos Guedes

**Affiliations:** 1 Laboratório de Imunologia Clínica, Instituto Oswaldo Cruz, Fundação Oswaldo Cruz—Fiocruz, Rio de Janeiro 21040-360, RJ, Brazil; patriciaamachado@micro.ufrj.br (P.d.A.M.); pollyannagomes@biof.ufrj.br (P.S.G.); 2 Laboratório de Imunobiotecnologia, Instituto de Microbiologia Paulo de Góes, Universidade Federal do Rio de Janeiro, Rio de Janeiro 21941-902, RJ, Brazil; monique@biof.ufrj.br; 3 Núcleo de Pesquisas em Parasitologia (NUPEP), Instituto de Ciências Biológicas, Universidade Federal de Juiz de Fora, Juiz de Fora 36036-900, MG, Brazil; 4 Laboratório de Imunofarmacologia, Instituto de Biofísica Carlos Chagas Filho (IBCCF), Universidade Federal do Rio de Janeiro, Rio de Janeiro 21941-902, RJ, Brazil; 5 Laboratório de Ultraestrutura Celular, Instituto Oswaldo Cruz, Fundação Oswaldo Cruz—Fiocruz, Rio de Janeiro 21040-360, RJ, Brazil; victor.midlej@ioc.fiocruz.br

**Keywords:** serine proteases, TPCK, leishmaniasis, *Leishmania amazonensis*, *Leishmania infantum*

## Abstract

Studies have previously demonstrated the importance of serine proteases in *Leishmania*. A well-known serine protease inhibitor, TPCK, was used in the present study to evaluate its in vitro and in vivo antileishmanial effects and determine its mechanism of action. Despite slight toxicity against mammalian cells (CC_50_ = 138.8 µM), TPCK was selective for the parasite due to significant activity against *L. amazonensis* and *L. infantum* promastigote forms (IC_50_ = 14.6 and 31.7 µM for *L. amazonensis* PH8 and Josefa strains, respectively, and 11.3 µM for *L. infantum*) and intracellular amastigotes (IC_50_ values = 14.2 and 16.6 µM for PH8 and Josefa strains, respectively, and 21.7 µM for *L. infantum*). *Leishmania* parasites treated with TPCK presented mitochondrial alterations, oxidative stress, modifications in lipid content, flagellar alterations, and cytoplasmic vacuoles, all of which are factors that could be considered as contributing to the death of the parasites. Furthermore, BALB/c mice infected with *L. amazonensis* and treated with TPCK had a reduction in lesion size and parasite loads in the footpad and spleen. In BALB/c mice infected with *L. infantum*, TPCK also caused a reduction in the parasite loads in the liver and spleen. Therefore, we highlight the antileishmanial effect of the assessed serine protease inhibitor, proposing a potential therapeutic target in *Leishmania* as well as a possible new alternative treatment for leishmaniasis.

## 1. Introduction

The *Leishmania* spp. complex is composed of protozoa that have *Phlebotomus* or *Lutzomyia* insects as the invertebrate hosts. These insects propagate the parasite when the female vector performs a blood meal on mammals, the vertebrate hosts, where the parasite life cycle continues [1]. Leishmaniasis can be categorized as cutaneous or visceral. Visceral manifestations can lead to death in more severe cases, while cutaneous manifestations can lead to irreversible disfigurement, causing social stigma [2]. The World Health Organization (WHO) [2], in its report on NTDs, ranked leishmaniasis as the second most important disease caused by a protozoan [2]. Annually, about 700,000 to 1 million new cases of leishmaniasis are registered, most of which are classified as the cutaneous clinical form [2]. According to the Pan-American Health Organization (PAHO) (2019), about two-thirds of the global burden of disease occurs in the Americas, and, of this total number of cases, 96% occur in Brazil [3].

*Leishmania* (*Leishmania*) *amazonensis* is an important etiological agent of human cutaneous leishmaniasis in the Americas [4]. However, studies have also reported some *L. amazonensis* isolates causing other clinical forms of the disease, including the visceral form [5]. One study reported that some patients from northeastern Brazil manifested rare and unusual visceral and diffuse forms due to infection by *L. amazonensis* [6]. *Leishmania* (*Leishmania*) *infantum* is the etiological agent of visceral leishmaniasis in South America, the Mediterranean basin, and West and Central Asia, with Brazil being the most affected country. Particularly in the Mediterranean, *L. infantum* can also cause cutaneous lesions. The visceral infection can result in impaired function of organs such as the liver, spleen, and bone marrow, with a fatal outcome in many cases [7].

The drugs used in leishmaniasis treatment have been the same for the past century [8] and are associated with major nephrotoxic, cardiotoxic, and hepatotoxic effects [9]. Thus, studies concerning the development of drugs against therapeutic targets not homologous to those of mammals and exclusively present in *Leishmania* parasites are highly important for clinical treatment. Preliminary studies have investigated the role of serine proteases of several protozoan infections [10] and assessed the use of serine protease inhibitors against these parasites, resulting in both specific and general modes of action that have successfully impaired parasite viability and/or reduced parasite virulence [10,11,12].

**Figure 1:**
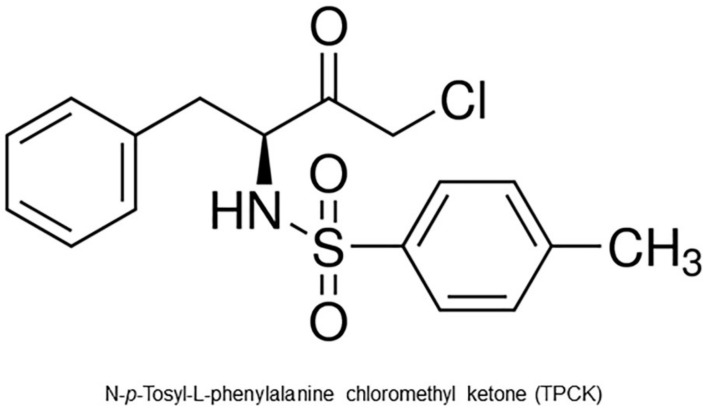
Chemical structure of N-*p*-Tosyl-L-phenylalanine cloromethyl ketone (TPCK).

N-*p*-tosyl-L-phenylalanine chloromethyl ketone (TPCK—Figure 1) is a serine protease inhibitor widely used in cell assays. It has been shown that TPCK significantly reduced *L. amazonensis* promastigote viability at 100 µM, with more evident effects compared to other protease inhibitors [13]. TPCK has also been reported as an NFκB inhibitor [14], with effects on several cell types [15], including in SARS-CoV experiments [16]. Furthermore, it also chemically modifies the side chain of His or Cys residues of the Nef protein critical for efficient viral replication and pathogenicity in HIV viruses [17]. TPCK is an irreversible serine protease inhibitor belonging to the chymotrypsin-like class that alkylates the His residue in the active site of serine proteases and has been widely applied in several parasite trials [11,18] and tested against other pathologies, such as cancer [19]. Although chymotrypsin is not present in the *Leishmania* genus [20], TPCK can inhibit other serine peptidases, as it has already been shown that TPCK can inhibit prolyl oligopeptidase of *L. infantum* [21]. In another study in which serine proteases were purified from *L. amazonensis*, a 68 kDa fraction was found to be 100% inhibited by TPCK [22]. In addition, subcellular fractions of 68 and ≥100 kDa from *L. amazonensis* were reported to have similar sensitivity to the inhibitory effect of TPCK, reaching between 50–80% inhibition rates [23]. Interestingly, no effect was observed when other protease inhibitors were used, suggesting that the action of TPCK on *L. amazonensis* is specific to serine proteases [23]. Subsequently, our group also demonstrated that TPCK displays an inhibitory capacity concerning purified serine proteases from the detergent fraction of *L. braziliensis* [24].

As yet, the TPCK effect against *Leishmania* has only been demonstrated in *L. amazonensis* promastigotes. In this context, we assessed the viability of *L. amazonensis and L. infantum* promastigotes and intracellular amastigotes following treatment with this compound for the first time, with the aim of determining the mechanism involved in the TPCK-induced death of the parasites. Furthermore, we assessed the in vivo effects of TPCK in murine models for both cutaneous and visceral leishmaniasis.

## 2. Results

### 2.1. Effect of TPCK on Peritoneal Macrophages and the Leishmania Parasites, L. amazonensis and L. infantum

**Figure 2:**
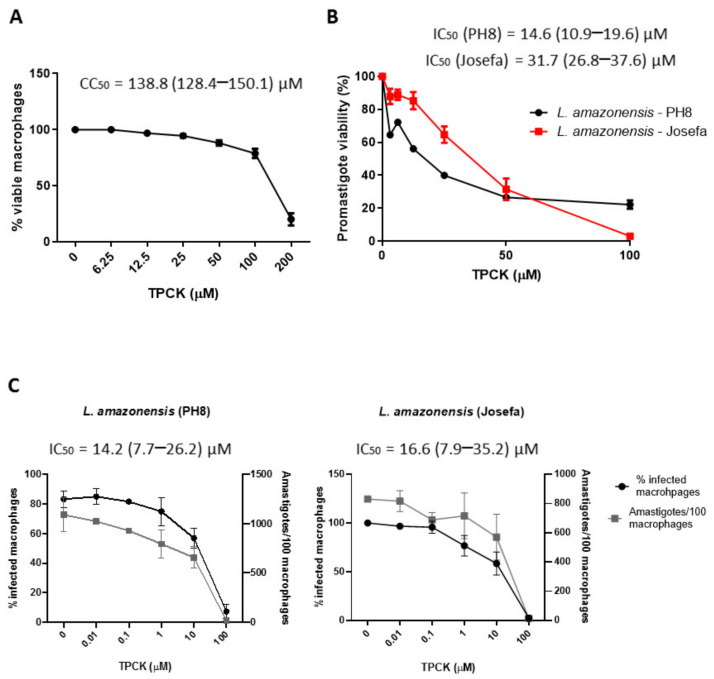
**In vitroTPCK effects against peritoneal macrophages and *L. amazonensis*.** (**A**) Cytotoxicity of TPCK against peritoneal macrophages from BALB/c mice. These cells were distributed in 96-well plates, incubated with TPCK at 37 °C under a 5% CO_2_ atmosphere for 72 h, and cell viability was determined by the MTT assay. (**B**) Inhibitory TPCK effect in *L. amazonensis* promastigotes (black line—PH8 strain, red line—Josefa strain). Promastigotes were distributed in 96-well plates, incubated with TPCK at 26 °C for 72 h, and cell viability was determined by the MTT assay. (**C**) Inhibitory TPCK effect in intracellular *L. amazonensis* amastigotes (PH8 and Josefa strains). These forms were treated with different TPCK concentrations for 72 h and subsequently counted. Graphs were plotted using GraphPad Prism 8 (GraphPad Software, Inc., La Jolla, CA, USA). Results are expressed as means ± standard error. IC_50_ and CC_50_ values were obtained by non-linear regressions using Graphpad Prism 8 software. Graphs are representative of three independent experiments.

Initially, TPCK toxicity towards mammalian cells was assessed. Figure 2A and Table 1 indicate that this compound presented slight toxicity, with a CC_50_ value of 138.8 µM, reducing cell viability by 80% at 200 µM and by 20% at 100 µM. In addition, TPCK did not cause hemolysis of human erytrocytes up to the maximum concentration tested (200 µM) (data not shown), which confirms the low in vitro toxicity of TPCK against mammalian cells.

The antileishmanial potential of TPCK on *L. amazonensis* (PH8 and Josefa strains) and *L. infantum* promastigotes and intracellular amastigotes was then evaluated. TPCK was active against the *L. amazonensis* promastigote forms, with IC_50_ values of 14.6 and 31.7 µM for the PH8 and Josefa strains, respectively (Figure 2B and Table 1). Antileishmanial activity was also observed against the amastigote form (IC_50_ values = 14.2 µM for PH8 and 16.6 µM for Josefa), reducing both the total number of amastigotes and the percentage of infected macrophages (Figure 2C and Table 1). Regarding TPCK selectivity, selectivity index (SI) values close to 10 were observed for *L. amazonensis* (PH8) and *L. amazonensis* (Josefa) amastigotes (Table 1). This indicates that this compound is about 10-fold more toxic to the parasite when compared to the host cell.

**Figure 3:**
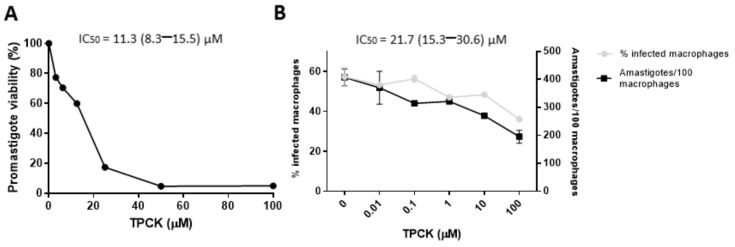
**In vitroTPCK effects against *L. infantum*.** (**A**) Inhibitory TPCK effect on *L. infantum* promastigotes. Promastigotes were distributed in 96-well plates, incubated with TPCK at 26 °C for 72 h, and cell viability was determined by the MTT assay. (**B**) Inhibitory TPCK effect on intracellular *L. infantum* amastigotes. These forms were treated with different TPCK concentrations for 72 h and subsequently counted. The graph shows the total number of amastigotes and the percentage of infected macrophages. Graphs were plotted using GraphPad Prism 8 (GraphPad Software, Inc., La Jolla, CA, USA). Results are expressed as means ± standard error. IC_50_ and CC_50_ values were obtained by non-linear regressions using Graphpad Prism 8 software. Graphs are representative of three independent experiments.

Likewise, TPCK displayed good antileishmanial efficacy against *L. infantum*, with IC_50_ values of 11.3 and 21.7 µM for promastigotes and amastigotes, respectively (Figure 3A,B and Table 1). It is interesting to highlight that TPCK was also selective against *L. infantum* amastigotes when compared to the host cells, with an SI value of 6.4 (Table 1). These data, therefore, demonstrate that although TPCK exhibits slight toxicity against host cells, it has a much greater effect on the two life cycle forms of *L. amazonensis* and *L. infantum*, displaying parasite selectivity.

### 2.2. Alterations Induced by TPCK in L. amazonensis and L. infantum

**Figure 4:**
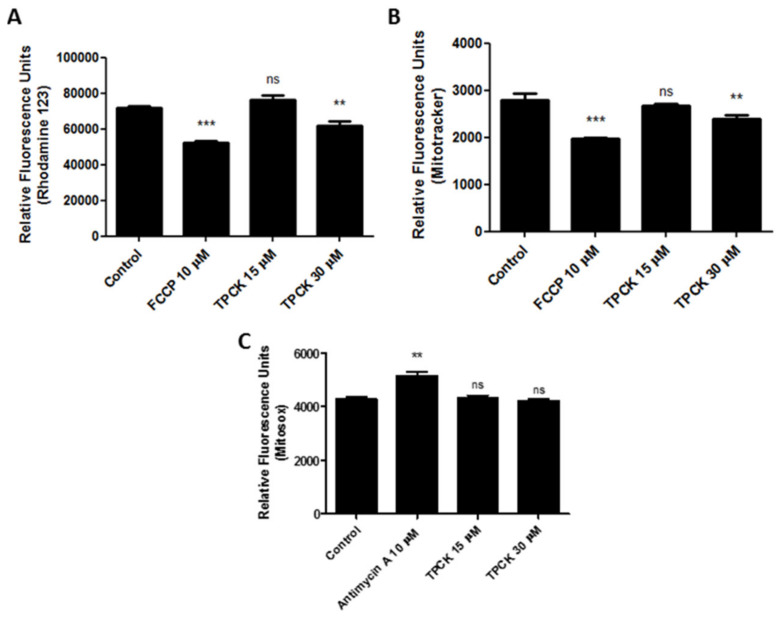
**TPCK treatment effects on the mitochondria of *L. amazonensis* promastigotes.***L. amazonensis* promastigotes were treated with TPCK (15 and 30 µM) for 24 h and then stained with either (**A**) rhodamine 123; (**B**) MitoTracker Red; or (**C**) MitoSox. TPCK concentrations correspond to the 1- and 2-fold IC_50_ values after 72 h of incubation. FCCP at 10 µM was used as the positive control in (**A**,**B**) and antimycin A at 10 μM in (**C**). Statistical analyses were performed by a one-way ANOVA followed by Tukey’s post-test to compare the untreated control group with the other treatments: *** *p* < 0.001, ** *p* < 0.01, and ns—not significant.

As TPCK displayed a good antileishmanial effect against *L. amazonensis* and *L. infantum*, we also evaluated some cellular parameters to determine the mode of action of this compound. Our studies started by assessing the mitochondria, as this organelle is unique in *Leishmania*, paramount to the survival of this parasite, and different from mammalian mitochondria. These facts make this organelle a potential drug target [25]. Treatment of *L. amazonensis* promastigotes with TPCK at 30 µM resulted in damage to parasite mitochondria, decreasing ΔΨ_m_ (despolarization) when compared with the untreated controls, as assessed by rhodamine 123 (Figure 4A; relative fluorescence units (RFU): ~74,000 in control and ~62,000 in 30 µM TPCK) and by MitoTracker Red staining (Figure 4B; RFU: ~2700 in control and ~2300 in 30 µM TPCK). FCCP (carbonyl cyanide-4-(trifluoromethoxy) phenylhydrazone) was used as the positive control and reduced ΔΨ_m_ in treated promastigotes, as observed by staining with both probes (Figure 4A,B). These data indicate that mitochondria may be a potential TPCK target in *L. amazonensis*. Interestingly, TPCK did not induce mitochondrial superoxide production, as observed by MitoSox staining, in treated *L. amazonensis* promastigotes (Figure 4C).

**Figure 5:**
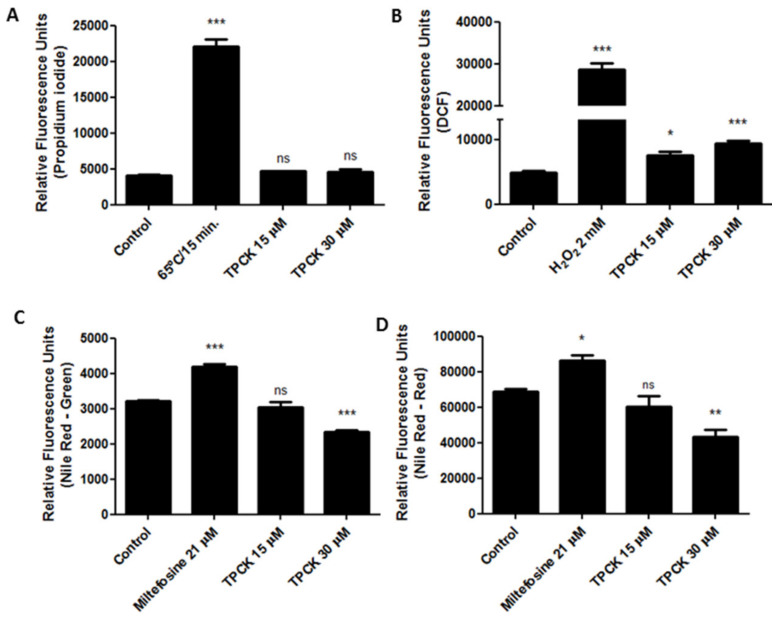
TPCK treatment effects on plasma membrane permeability, oxidative stress, and lipid content of *L. amazonensis* promastigotes. *L. amazonensis* promastigotes were treated with TPCK (15 and 30 µM) for 24 h and stained with either (**A**) propidium iodide; (**B**) H_2_DCFDA; (**C**) Nile Red (green fluorescence); and (**D**) Nile Red (red fluorescence). TPCK concentrations correspond to the 1- and 2-fold IC_50_ values after 72 h of incubation. Promastigotes heated at 65 °C for 15 min were used as the positive controls in (**A**); 2 mM H_2_O_2_ in (**B**); and 21 µM miltefosine in (**C**,**D**). Statistical analyses were performed by a one-way ANOVA followed by Tukey’s post-test to compare the untreated control group with the other treatments: *** *p* < 0.001, ** *p* < 0.01, * *p* < 0.05, and ns—not significant.

In addition, plasma membrane permeability after treatment with TPCK was assessed by propidium iodide (PI) staining. Figure 5A indicates that this compound did not alter plasma membrane permeability of *L. amazonensis* promastigotes at both tested concentrations when compared to control cells because the RFUs were similar between these groups. Promastigotes heated to 65 °C for 15 min were used as positive controls, exhibiting significantly altered plasma membrane permeability (Figure 5A).

Treatment with TPCK also caused oxidative stress in *L. amazonensis* promastigotes, as verified through H_2_DCFDA staining. Cells treated with TPCK (15 and 30 µM) displayed increased fluorescence intensity for DCF when compared to untreated controls (Figure 5B; RFU: ~4900 in control; ~7500 in 15 µM TPCK and ~9300 in 30 µM TPCK). H_2_O_2_ (2 mM) was used as the positive control and also exhibited increased fluorescence intensity for DCF (Figure 5B). This TPCK-caused oxidative stress may have been a contributor to the cell death of *L. amazonensis* promastigotes.

Interestingly, decreased lipid content in *L. amazonensis* promastigotes treated with TPCK at 30 µM was noted (Figure 5C,D). This was observed for both neutral lipids (readings taken at 485/528 nm—green fluorescence) (Figure 5C; RFU: ~3200 in control and ~2300 in 30 µM TPCK) and polar lipids/phospholipids (readings taken at 540/600 nm—red fluorescence) (Figure 5D; RFU: ~69,000 in control and ~43,000 in 30 µM TPCK), demonstrating that the lipid content is significantly altered after TPCK treatment. Miltefosine (21 µM) was used as a positive control and increased the Nile Red fluorescence in both readings (Figure 5C,D).

**Figure 6:**
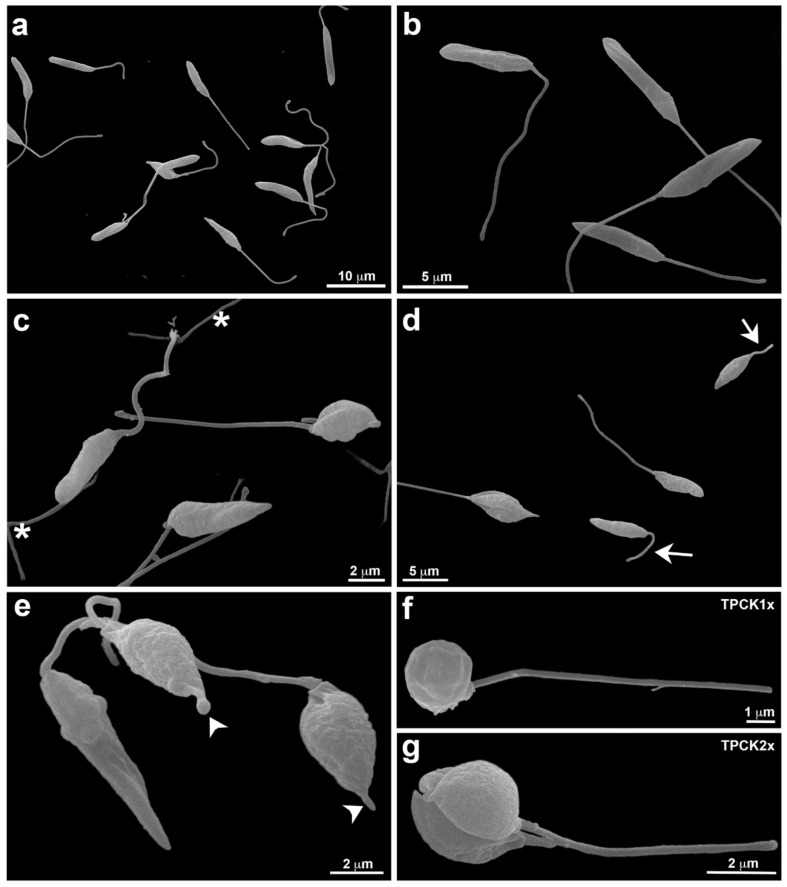
**Morphology of *L. infantum* promastigotes treated with TPCK, as observed using SEM.** ((**a**,**b**) Control (no treatment), note the regular cell morphology; (**c**) promastigotes treated with TPCK at 12 µM displaying parasite cell body alteration and loose flagella portions in the background (asterisks); (**d**,**e**) promastigotes treated with TPCK at 24 µM, parasite cell body changes can also be observed and short flagella are visualized (**d**—arrow). A membrane projection in the anterior body portion is noted (**e**—arrowhead); (**f**) promastigotes treated with TPCK at 12 µM; (**g**) promastigotes treated with TPCK at 24 µM.

Figure 6 displays the morphological analysis of *L. infantum* promastigotes treated with TPCK (12 and 24 µM), as evaluated by scanning electron microscopy (SEM). Untreated cells with fusiform elongated morphology and flagella longer than the cell body are presented in Figure 6a,b. After treatment with 12 µM TPCK, cells appeared less fusiform and more conical and rounded (Figure 6c). Furthermore, loose flagella portions could be noted (Figure 6c). TPCK at 24 µM also induced the appearance of conical and rounded parasites with reduced cell volume, while parasites with a short flagellum were also observed (Figure 6d). Treatment with 24 µM TPCK induced the appearance of cells with membrane projections in the anterior portion (Figure 6e). Figure 6f,g indicate cells exhibiting completely rounded morphology for both TPCK treatment concentrations (Figure 6f, 12 µM and Figure 6g, 24 µM).

**Figure 7:**
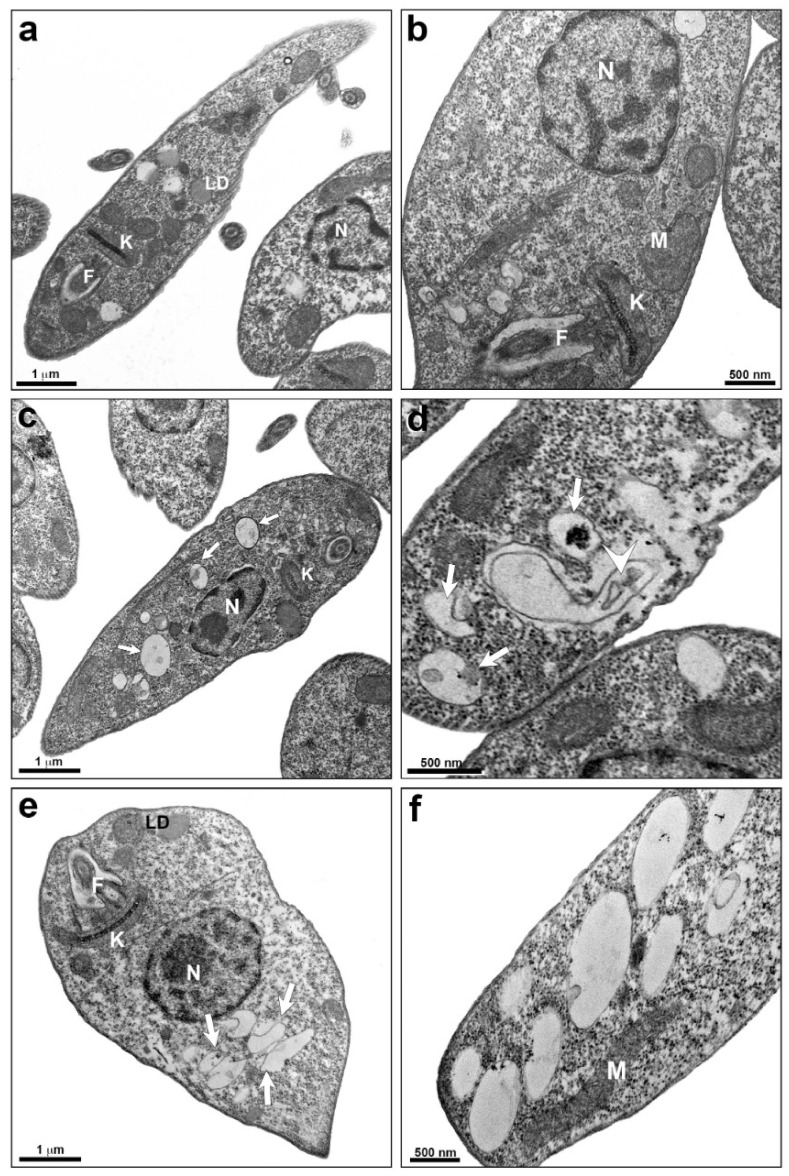
**Ultrastructural analysis of *L. infantum* promastigotes treated with TPCK, as observed by TEM.** (**a**,**b**) Control (untreated cells), note the regular organelle aspect and homogeneous cytoplasm; (**c**,**d**) promastigotes treated with TPCK at 12 µM displaying an increased number of cytoplasmatic vacuoles (**c**—arrow) and a membranous profile inside some vacuoles (**d**—arrowhead); (**e**,**f**) promastigotes treated with TPCK at 24 µM, several vacuoles can be observed (**e**—arrow). N, nucleus; F, flagellum; K, kinetoplast; M, mitochondria; LD, lipid droplets.

An ultrastructural analysis of *L. infantum* promastigotes treated with TPCK was also performed by transmission electron microscopy (TEM). Untreated cells present some lipid bodies, a regular kinetoplast morphology, and homogeneous cytoplasm (Figure 7a,b). Cells treated with TPCK at 12 µM presented an increased number of cytoplasmic vacuoles (Figure 7c) and some vacuoles with membranous material (Figure 7d). Treatment with TPCK at 24 µM likewise induced the appearance of cytoplasmic vacuoles (Figure 7e,f).

**Figure 8:**
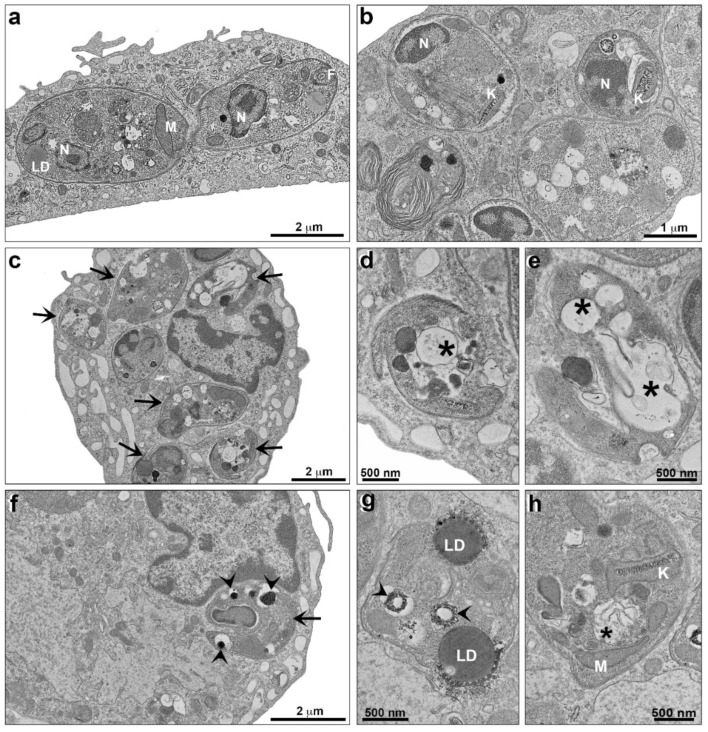
**Ultrastructural analysis of *L. infantum* intracellular amastigotes treated with TPCK observed by TEM.** (**a**,**b**) Control (untreated cells), amastigotes are seen inside parasitophorous vacuoles with a homogeneous cytoplasm and regular organelle aspects; (**c**–**e**) treatment with TPCK at 22 µM, amastigotes are indicated with arrows (**c**). Large cytoplasmatic vacuoles with membranous profiles can be visualized (**d**,**e**—asterisks); (**f**–**h**) treatment with TPCK at 44 µM, an amastigote is indicated with an arrow (**f**). Electron dense material (**g**—arrowhead) and a membranous profile (**h**—asterisks) can be observed inside vacuoles. N, nucleus; LD, lipid droplets; M, mitochondria; K, kinetoplast; F, flagellum.

Concerning *L. infantum* and *L. amazonensis* intracellular amastigotes, treatment with TPCK also caused ultrastructural changes. Macrophages that were infected with *L. infantum* and left untreated (control) presented amastigotes inside parasitophorous vacuoles, with the parasites displaying homogeneous cytoplasm and kinetoplasts and mitochondria with regular morphology, as well as small cytoplasmic vacuoles (Figure 8a,b). Infected macrophages treated with 22 µM TPCK presented morphological changes in the amastigotes (Figure 8c), including large cytoplasmic vacuoles (Figure 8d,e) with membranous profiles (Figure 8e). Treatment of infected macrophages with 44 µM TPCK resulted in the appearance of amastigotes with cytoplasmic vacuoles containing electron-dense material (Figure 8f,g) and vacuoles containing membrane profiles (Figure 8h).

**Figure 9:**
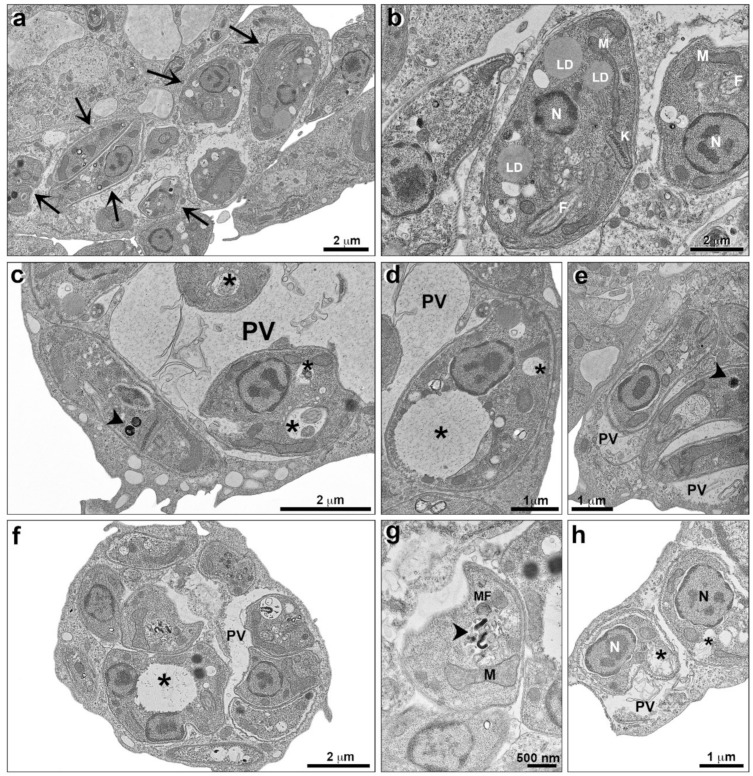
**Ultrastructural analysis of *L. amazonensis* intracellular amastigotes treated with TPCK observed by TEM.** (**a**,**b**) Control amastigotes (untreated) are seen inside parasitophorous vacuoles (arrows) with a homogeneous cytoplasm and regular organelle aspects; (**c**–**e**) treatment with TPCK at 15 µM, a parasitophorous vacuole extension inside macrophages is noted. Large amastigote cytoplasmatic vacuoles are seen (**c**,**d**—asterisks), some containing a membranous profile (**e**—arrowhead) or electron dense material (**c**—arrowhead); (**f**–**h**) treatment with TPCK at 30 µM, electron-dense material (**g**—arrowhead) and membranous profile inside large vacuoles (**h**—asterisks) are observed. N, nucleus; LD, lipid droplets; M, mitochondria; K, kinetoplast; PV, parasitophorous vacuole; MF, myelinic figures.

Macrophages infected with *L. amazonensis* and left untreated presented amastigotes inside parasitophorous vacuoles, with parasite organelles exhibiting regular morphology (Figure 9a,b). After treatment with TPCK at 15 µM, membrane material was observed within the parasitophorous vacuole (Figure 9c). Furthermore, large vacuoles in the parasite cytoplasm containing granular material (Figure 9c,d) and the presence of electron-dense material (Figure 9c,e) were also observed. Treatment with 30 µM TPCK caused more damage to amastigotes than the lower concentration, and cytoplasmic vacuoles containing granular material were also observed within these treated amastigotes (Figure 9f,h). Concentric membrane profiles forming myelinic figures were noted (Figure 9g). Figure 8h indicates parasites with ruptured plasma membranes. Vacuoles were also observed in the cytoplasm of macrophages infected and treated with TPCK.

### 2.3. In Vivo Treatment with TPCK

**Figure 10:**
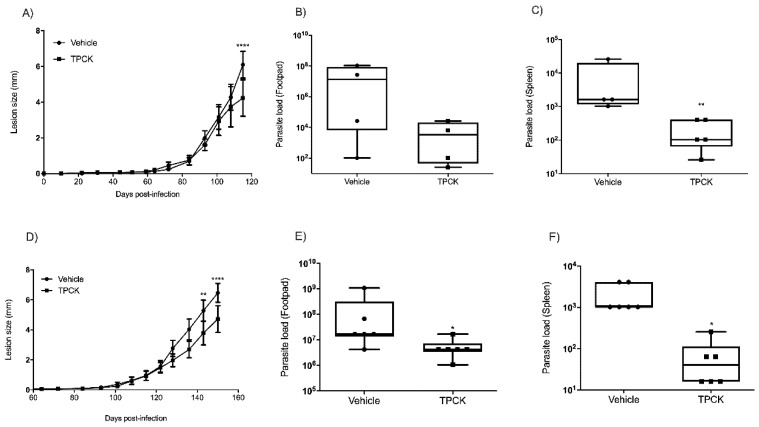

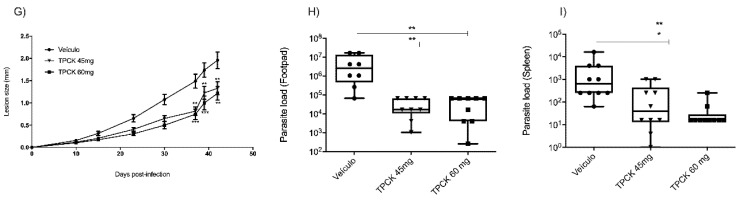
**In vivo TPCK effect in a murine model for cutaneous leishmaniasis**. BALB/c mice were infected in the right hind paw with 2 × 10^6^ *L. amazonensis* promastigotes (Josefa strain) and treated with TPCK at 15, 30, 45, and 60 mg/kg intraperitoneally, three times per week, for a total of 10 doses. (**A**) Lesion sizes (mm): vehicle and 15 mg/kg TPCK, were measured once weekly. (**B**) Parasite loads in footpads: vehicle and 15 mg/kg TPCK. (**C**) Parasite loads in spleens: vehicle and 15 mg/kg TPCK. (**D**) Lesion sizes (mm): vehicle and 30 mg/kg TPCK were measured once weekly. (**E**) Parasite loads in footpads: vehicle and 30 mg/kg TPCK. (**F**) Parasite loads in spleens: vehicle and 30 mg/kg TPCK. (**G**) Lesion sizes (mm): vehicle and 45 and 60 mg/kg TPCK were measured once weekly. (**H**) Parasite loads in footpads: vehicle and 45 and 60 mg/kg TPCK. (**I**) Parasite loads in spleens: vehicle and 45 and 60 mg/kg TPCK. Statistical differences between mean values were evaluated by the non-parametric Student *t*-test: * *p*  ≤  0.05, ** *p* < 0.01, *** *p* < 0.001, **** *p* < 0.0001.

The in vivo effect of TPCK treatment of *L. amazonensis- or L. infantum-*infected animals was assessed for the first time. BALB/c mice were infected in the right hind footpad with 2 × 10^6^ *L. amazonensis* promastigotes in the stationary growth phase. TPCK treatment commenced when the lesions began to appear (Δ = 0.5 mm). Doses were administered three times a week, totaling 10 doses. As presented in Figure 10, groups were treated with 15, 30, 45, and 60 mg/kg intraperitoneally and the negative control group comprised mice treated with castor oil. At 15 mg/kg TPCK, lesion sizes did differ statistically from the control group (Figure 10A), but parasite load in footpad and spleen did not differ statistically in comparison to the control group (Figure 10B,C). Animals treated with TPCK at 30, 45, and 60 mg/kg displayed reduced injury in the chronic phase of the disease, with decreased lesion sizes (Figure 10D,G) and parasite loads at the infection site (Figure 10E,H). In addition, treated mice also presented reduced parasite loads in the spleen (Figure 10F,I), indicating partial prevention of visceralization.

**Figure 11:**
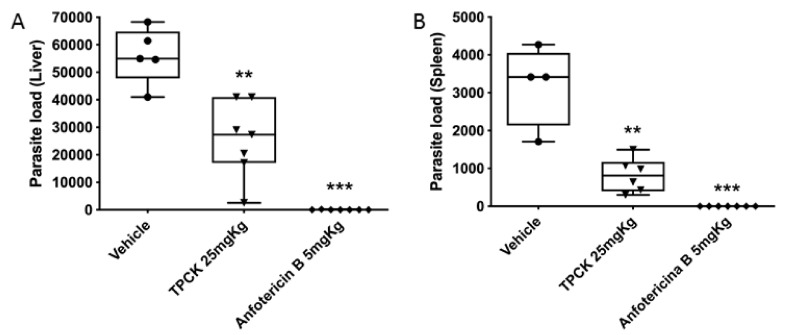
**In vivo TPCK effect in a murine model for visceral leishmaniasis**. BALB/c mice were infected intraperitoneally with 2.5 × 10^7^ stationary-phase *L. infantum* promastigotes. Seven days after infection, the animals were treated with TPCK at 25 mg/kg/day intraperitoneally for 10 days. The control group received vehicle (PBS-Tween 80 0.05%). (**A**) Parasite load in the liver (parasites/liver). (**B**) Parasite loads in the spleen (parasites/spleen). Statistical differences between mean values were evaluated by the non-parametric Student *t*-test: ** *p* < 0.01, *** *p* < 0.001.

For the visceral murine model, mice were intraperitoneally infected with 2.5 × 10^7^ *L. infantum* promastigotes in the stationary growth phase, and treatment commenced on day 7 post-infection. Intraperitoneal doses of 25 mg/kg TPCK were administered daily for a total of 10 doses. Figure 11 shows that the treatment with TPCK intraperitoneally reduced the parasite load in the liver (Figure 11A) and spleen (Figure 11B). The negative control group comprised infected mice treated with vehicle (PBS-Tween 80). Animals treated with amphotericin B (5 mg/kg) showed a great reduction in the parasite load of the liver and spleen (Figure 11A,B).

In vivo TPCK toxicity was also evaluated in the present study. TPCK did not significantly alter creatinine, AST (aspartate aminotransferase), and ALT (alanine aminotransferase) levels in the serum of *L. amazonensis-* (Figure S1) or *L. infantum-*infected animals (Figure S2). This indicates that TPCK did not induce renal and hepatic toxicity in treated animals when compared to the untreated infected animals (control group).

## 3. Discussion

Proteases are known to be important for several regulatory functions, including physiological processes (programmed cell death), stress responses (heat shock and anoxia), cell–cell recognition, and signal translation [26,27,28]. TPCK is a specific inhibitor of chymotrypsin-like serine proteases and is very effective in preventing proteolytic activity [29].

In fungal infections, it was reported that TPCK could prevent pathogen replication [11], while Trivedi and coworkers demonstrated that TPCK acts in a dose-dependent manner to prevent the viral replication of HIV-1 [30]. Furthermore, TPCK has also been shown to block the development of dipteran *Oxysarcodexia thornax* larvae. These larvae homogenates contain a complex proteolytic profile, ranging from 21.5 to 136 kDa, and TPCK was able to completely inactivate all enzyme activities from the homogenates [31].

A previous study demonstrated the importance of serine protease inhibitors in blocking *Plasmodium* merozoite cell egress [32]. Studies with *L. donovani* using different classical serine protease inhibitors (aprotinin, TPCK, benzamidine, and anti-serine protease antibodies) have demonstrated that aprotinin appears to be more potent in arresting promastigote growth, with significant morphological alterations [18]. Moreover, natural protease inhibitors have been identified in *L. donovani*, suggesting that this mechanism is essential for maintaining the development cycle even in invertebrate hosts [33].

In this work, TPCK showed an antileishmanial effect against both promastigote and amastigote forms of *L. amazonensis* and *L. infantum*, showing potency from 14.2 to 21.7 at the relevant stage (amastigote forms) and selectivity close to 10. Previously, Silva-Lopez and colleagues showed that TPCK at 100 µM significantly reduced the viability of *L. amazonensis* promastigotes by about 63% after 8 h of incubation, 82% after 16 h, and 93% after 32 h [13]. TPCK has already been shown to reduce serine protease activity of *L. amazonensis* [34]. Interestingly, PF-429242, a serine protease inhibitor previously studied by our group, was active against both *L. infantum* promastigotes (IC_50_ = 2.78 μM) and amastigotes (IC_50_ = 14.07 μM), displaying parasite selectivity over the host cell [35].

One of the major issues related to current leishmaniasis chemotherapy is the toxicity of the available drugs, which can cause several adverse side effects, thus making the discovery of new compounds that exhibit low toxicity paramount [36]. In this work, TPCK showed low toxicity in mammalian cells and was found to be selective for the parasite when compared to host cells. Interestingly, our research group has previously demonstrated that PF-429242 also exhibits low toxicity against peritoneal macrophages [35].

In the present work, *Leishmania* parasites treated with TPCK presented mitochondrial alterations, oxidative stress, modifications in lipid content, flagellar alterations, and cytoplasmic vacuoles, factors that may be contributing to the death of the parasites. In *Candida parapsilosis*, TPCK also induced ROS production while increasing sterol and neutral lipid contents [11]. It is well known that ROS cause damage to lipids, proteins, nucleic acids, and other important biosystem components [37]. It is interesting to highlight that increased ROS levels are frequently associated with lipid generation, with increased lipid contents and lipid droplet accumulation [37,38], which has already been reported for *Leishmania* parasites treated with different compounds [39,40]. However, the opposite was observed herein, as increased ROS levels and decreased lipid contents were detected in TPCK-treated *L. amazonensis* promastigotes. This suggests that the occurrence of these events in *L. amazonensis* following TPCK treatment may be unrelated and induced directly by the compound. Decreased cellular lipid content reported in TPCK-treated muscle cells has been suggested to be due to NF-κB inhibition [15].

Previously, Silva-Lopez and colleagues evaluated the ultrastructure of *L. amazonensis* promastigotes treated with TPCK and observed alterations in the flagellar pocket region, bleb plasma membrane formation, and increased intracellular vesicular bodies [13]. Ultrastructural alterations induced by TPCK in amastigote forms, however, are described for the first time in the present study. The alterations observed by Silva-Lopez and colleagues are compatible with those reported herein for *L. infantum* promastigotes and amastigotes, as well as in *L. amazonensis* amastigotes; these include flagellar alterations and cytoplasmic vesicle formation, with further modifications such as oxidative stress and mitochondrial and lipid alterations.

It is interesting to note that PF-429242 and TPCK, two protease inhibitors, induced different cellular change patterns in *Leishmania*. PF-429242 was shown to cause a significant mitochondrial alteration, with ΔΨ_m_ hyperpolarization and superoxide production and neutral lipid accumulation. TPCK also induced mitochondrial damage, but to a lesser extent when compared to PF-429242, and unlike PF-429242 treatment, TPCK decreased lipid content. Both inhibitors induced the appearance of cell cytoplasmic vesicles, caused flagellar changes, and induced oxidative stress [35].

Various mitochondrial proteases may be involved in mitochondrial homeostasis and functioning [41,42,43]. Thus, as PF-429242 and TPCK have different implications for the mitochondrial status, these compounds may inhibit different *Leishmania* mitochondrial proteases.

Subtilisin-deficient *L. donovani* amastigotes display a retained flagellum [44]. Interestingly, PF-429242 and TPCK caused flagellar changes in *Leishmania*, which may be triggered by this serine protease inhibition [35].

The in vivo effects of TPCK against cutaneous and visceral murine leishmaniasis were assessed in this work for the first time. TPCK treatment caused a significant reduction in lesion size and parasite load of animals infected with Leishmania. In a hypoxic ischemic model study using neonatal rats treated intraperitoneally with TPCK, decreased ischemic lesions were observed [45], which was similar to that reported by Hara and coworkers in adult gerbils [46]. Furthermore, TPCK significantly attenuated inflammatory parameters in a rheumatoid arthritis model in vivo by reducing the production of proinflammatory cytokines [47].

*Leishmania* serine proteases can be considered potential therapeutic targets for leishmaniasis treatment due to their involvement in crucial cellular functions such as host cell infection, promastigote differentiation into amastigotes, proliferation inside macrophages, virulence, and resistance to oxidative damage [48]. Several research groups have been motivated to study protease inhibitors that target these important *Leishmania* virulence factors as therapeutic alternatives [48]. In this work, we demonstrate the great effect of TPCK on *L. infantum* and *L. amazonensis* in vitro and in vivo. In addition, we also demonstrate that treatment of promastigotes and amastigotes with this protease inhibitor causes damage to the parasite mitochondria, oxidative stress, alteration of lipid content, morphological and flagellar changes, as well as an increase in cytoplasmic vesicles, often containing electron-dense material or membrane profiles.

The development of local therapies has gained prominence in the treatment of leishmaniasis as a way to avoid the toxicity and discomfort of systemic therapy. This could be an interesting proposal for formulations containing TPCK [49]. Nanoparticles, meanwhile, can enhance the pharmacokinetic properties of the active principle, improve bioavailability, protect against compound degradation, and reduce toxicity, targeting compound delivery directly into the macrophage [48]. Considering that TPCK displays important in vitro and in vivo parasite growth inhibition activity, we propose delivery improvements as a future perspective in order to reach deeper dermis layers through topical formulations or nanoparticles to direct to macrophages.

In conclusion, the in vitro and in vivo antileishmanial effects of the serine protease inhibitor, TPCK, against *L. amazonensis* and *L. infantum* were reported herein, confirming the importance of serine proteases as targets for potential leishmaniasis therapies.

## 4. Material and Methods

### 4.1. Parasites

Two *L. amazonensis* strains were used: MHOM/BR75/Josefa and IFLA/BR/1967/PH8. *L. infantum* (MHOM/MA/67/ITMAP-263) was also used. The promastigotes were maintained in a BOD incubator (MOD. 347 CD; FANEM, São Paulo, Brazil) at 26 °C in RPMI 1640 medium (Sigma-Aldrich, St. Louis, MO, USA) supplemented with 10% fetal bovine serum (FBS), 100 μg/mL streptomycin, 100 U/mL penicillin, 5 mg/mL hemin, 0.5 mg/mL folic acid, 0.2 mg/mL D-biotin, and 4 mg/mL adenine. To maintain virulence, the parasites were routinely obtained from infected BALB/c mice, and all tests were performed with parasites that had been cultured to a maximum of three in vitro passages.

### 4.2. Mice

Six- to eight-week-old BALB/c mice were obtained from the Central Animal Facility of the Universidade Federal do Rio de Janeiro (UFRJ) and Universidade Federal de Juiz de Fora (UFJF). All procedures for the use and maintenance of animals were performed according to protocols approved by the Ethical Committee for Animal Handling (CEUA 080/2018 from UFRJ and 008/2018 from UFJF).

### 4.3. Chemicals

TPCK was purchased from Sigma-Aldrich, ≥97% purity, and diluted in dimethyl sulfoxide (DMSO). The highest DMSO concentration used in the tests was 0.03%, which is non-toxic to mammalian cells or parasites. DMSO was also purchased from Sigma-Aldrich, purity ≥99.9%, as was Tween^®^80. Amphotericin B (Cristália, Itapira, São Paulo, Brazil), diluted in deionized water, was used as a reference drug in the antileishmanial tests against promastigotes and amastigotes.

### 4.4. Cytotoxicity Assay

To assess TPCK toxicity in mammalian cells, peritoneal macrophages from BALB/c mice, obtained by peritoneal lavage according to Layoun and coworkers, with modifications, were used [50]. The cells were distributed in 96-well plates at 2 × 10^6^ cells/mL in RPMI medium containing 10% FBS and a 0.5% penicillin and streptomycin solution (Sigma-Aldrich). The plates were incubated for 1 h at 37 °C under a 5% CO_2_ atmosphere, followed by washing three times with phosphate-buffered saline (PBS) and then re-incubated in medium overnight. The macrophages were washed again with PBS, and TPCK was added (0–200 µM) for 72 h. After this period, 3-(4,5-dimethylthiazol-2-yl)-2,5-diphenyltetrazolium bromide (MTT—Sigma-Aldrich) was added at a concentration of 5 mg/mL, and the plate was incubated for 2 h at 37 °C and 5% CO_2_. Next, an isopropanol/HCl solution (0.7%) was added to stop the reaction, and absorbances were determined using a spectrophotometer (Multiskan EX, Thermo Fisher Scientific, Waltham, MA, USA) at 570 nm. CC_50_ values were determined using the GraphPad Prism 8 software (San Diego, CA, USA). All assays were performed in duplicate, comprising three independent experiments. A 2% Triton X-100 solution was used as a positive control and killed 93.36% of macrophages.

### 4.5. Anti-Promastigote Activity

*L. amazonensis* (PH8 and Josefa strains) or *L. infantum* promastigote forms in the log phase of growth were distributed in 96-well plates at 3 × 10^6^ cells/mL, and TPCK at concentrations from 0 to 100 µM was added. Promastigotes maintained in the RPMI medium containing FBS and antibiotics were used as the control group. After 72 h at 26 °C, MTT was added and incubated for 4 h. As previously described, the reaction was stopped by adding an isopropanol/HCl solution, and the reading was performed. Percentage growth inhibition rates were then calculated compared to the non-treated control, and IC_50_ values were determined using GraphPad Prism 8 software. All assays were performed in duplicate, comprising three independent experiments.

### 4.6. Anti-Amastigote Activity

Peritoneal macrophages from BALB/c mice obtained by peritoneal lavage were distributed over 13 mm glass coverslips in 24-well plates at 2 × 10^6^ cells/mL in RPMI medium containing 10% FBS and a 0.5% penicillin and streptomycin solution. The plates were then incubated for 1 h at 37 °C and 5% CO_2_, washed three times with PBS and then re-incubated in medium overnight. The cells were washed again with PBS and infected with stationary growth phase *L. amazonensis* promastigotes (PH8 and Josefa strains) at a 5:1 ratio for 4 h at 33 °C or with stationary growth stationary phase *L. infantum* promastigotes at a 10:1 ratio for 24 h at 37 °C. Subsequently, each well was washed with PBS, and TPCK was added at different concentrations (0–100 µM) in RPMI medium containing 10% FBS and a 0.5% penicillin and streptomycin solution. Control wells did not receive treatment, only RPMI medium containing FBS and a penicillin and streptomycin solution. All assays were performed in triplicate. After 72 h of treatment, the coverslips were removed, and the cells were stained with Panotico. The coverslips were then mounted on slides, and a total of 100 macrophages were counted for each coverslip. Results were calculated as the percentage of amastigote growth inhibition compared to the non-treated control group, and IC_50_ values were determined using GraphPad Prism 8 software. Three independent experiments were performed for each strain and species. The total number of amastigotes and the percentage of infected macrophages were also calculated.

### 4.7. Mitochondrial Membrane Potential (ΔΨ𝑚) Determination

The ΔΨ𝑚 of *L. amazonensis* promastigotes (PH8) treated with TPCK (15 and 30 µM—approximate values to the 1- and 2-fold *L. amazonensis* promastigote IC_50_ values after 72 h incubation) for 24 h was evaluated using rhodamine 123 (Sigma-Aldrich) and MitoTracker^®^ Red CM-H2XRos (Life Technologies, Carlsbad, CA, USA). These stainings were performed as described by Machado and coworkers (2021) [35]. The negative control comprised promastigotes maintained in RPMI medium with FBS and antibiotics. FCCP (Sigma-Aldrich) at 10 µM was used as a positive control in both cases, and three independent experiments were performed in triplicate for each probe.

### 4.8. Mitochondrial Superoxide Production Determination

The superoxide levels in *L. amazonensis* promastigotes (PH8) treated with TPCK (15 and 30 µM) were determined after a 24 h incubation period. The test was performed as described by Machado and coworkers (2021) [35]. Promastigotes maintained in RPMI medium with FBS and antibiotics were used as the negative controls and those treated with antimycin A (Sigma-Aldrich) at 10 μM were used as positive controls. Three independent experiments were performed in triplicate.

### 4.9. Plasma Membrane Permeability Assessments

After treatment with TPCK (15 and 30 µM) for 24 h, *L. amazonensis* (PH8) promastigotes were labeled with propidium iodide (PI), obtained from Sigma-Aldrich, as described by Machado and coworkers (2021) [35]. Positive controls were obtained by incubating promastigotes at 65 °C for 15 min, and negative controls were obtained following promastigote maintenance in RPMI medium containing FBS and antibiotics. All assays were performed in triplicate, comprising three independent experiments.

### 4.10. Intracellular Reactive Oxygen Species (ROS) Level Determinations

After treatment of *L. amazonensis* (PH8) promastigotes with TPCK (15 and 30 µM), promastigotes were labeled with H_2_DCFDA (Invitrogen, Waltham, MA, USA), as described by Machado and coworkers (2021) [35]. Promastigotes treated with 2 mM H_2_O_2_ were used as the positive control of this assay as H_2_O_2_ is an ROS and directly reacts with the H_2_DCFDA probe, leading to conversion to DCF, a highly fluorescent compound. Promastigotes maintained in RPMI medium containing FBS and antibiotics were used as the negative controls. The assay was performed in triplicate, comprising three independent experiments.

### 4.11. Lipid Accumulation Assessments

After treatment with TPCK (15 and 30 µM), 1 × 10^7^ promastigotes/mL *L. amazonensis* (PH8) in 200 µL were incubated with Nile Red (Sigma-Aldrich) at 10 µg/mL for 30 min at 26 °C. Fluorescence intensity was evaluated at 485/528 and 540/600 nm excitation/emission wavelengths, respectively. Promastigotes treated with miltefosine (21 µM) were used as positive controls, and those maintained in RPMI medium with FBS and antibiotics comprised the negative controls. Three independent experiments were performed in triplicate.

### 4.12. Scanning Electron Microscopy (SEM)

*L. infantum* promastigotes treated with TPCK (12 and 24 µM—approximate values to 1- and 2-fold *L. infantum* promastigote IC_50_ values after a 72 h incubation) for 24 h were spread on coverslips treated with 0.01% poly-L-lysine and fixed in 2.5% glutaraldehyde in a 0.1 M sodium cacodylate buffer at room temperature for 1 h. The promastigotes were post-fixed in 1% osmium tetroxide (OsO_4_) for 15 min and dehydrated in an increasing concentration series of ethanol (7.5, 15, 30, 50, 70, 90, and 100%) for 15 min in each step. The samples were critical point dried with CO_2_, sputter-coated with a 15 nm thick layer of gold, and observed on a JEOL JSM 6390 scanning electron microscope (Tokyo, Japan). Promastigotes maintained in the culture medium were used as a negative control.

### 4.13. Transmission Electron Microscopy (TEM)

After TPCK treatment of *L. infantum* promastigotes (12 and 24 µM) and peritoneal macrophages infected with either *L. amazonensis* amastigotes (15 and 30 µM) or *L. infantum* amastigotes (22 and 44 µM), the samples were fixed in 2.5% glutaraldehyde in 0.1 M sodium cacodylate buffer at room temperature for 1 h and post-fixed in 1% OsO_4_ and 0.8% potassium ferrocyanide solution. The cells were dehydrated using an increasing concentration series of acetone (70, 90, and 100%), embedded in Epon resin, and polymerized at 60 °C. Ultrathin sections (60–70 nm thick) were stained with 5% uranyl acetate and lead citrate and examined using a Hitachi HT 7800 transmission electron microscope (Ibaraki, Japan). Cells maintained in culture medium were used as a negative control. The concentrations used correspond to 1- and 2-fold the IC_50_ values in promastigotes or amastigotes after a 72 h incubation period.

### 4.14. In Vivo Assay and Treatment

Six-to-eight-week-old female BALB/c mice were subcutaneously infected in the right hind footpad with 2 × 10^6^ stationary-phase *L. amazonensis* promastigotes (Josefa strain). The course of the infection was monitored by measuring increases in footpad thickness with a dial caliper. The treatment was performed by daily intraperitoneal injections of 15, 30, 45, and 60 mg/kg TPCK for a total of 10 doses, and the non-treated control animal group received a vehicle (castor oil).

For the visceral murine model, six-to-eight-week-old female BALB/c mice were intraperitoneally infected with 2.5 × 10^7^ stationary-phase *L. infantum* promastigotes. Seven days after infection, the treatment was initiated and performed with 10 doses daily of 25 mg/kg, and the non-treated control animal group received a vehicle (PBS with 0.05% Tween 80).

### 4.15. Limiting Dilution Assay (LDA)

The infected footpads were excised and placed for 1 min in 70% alcohol for disinfection. Liver and spleen were also removed and placed in Eppendorfs containing 1 mL of 199 medium with 10% FBS. Footpads, livers, and spleens were homogenized. In 96-well plates, 50 μL of the homogenates were serially diluted four-fold in 150 μL of 199 medium per well. The plates were incubated in a BOD incubator at 26 °C for 7 to 14 days. At the end of this period, the plates were visually evaluated under an optical microscope, and the last well in which parasites could be seen was considered in the determination of the parasite load. Thus, determining the parasite load was as follows: Number of parasites = 4X/(mass of organs in grams), where X is the number of the last well in which parasites were observed.

### 4.16. Statistical Analyses

Statistical analyses were performed using GraphPad Prism 8 software (GraphPad Software, Inc., La Jolla, CA, USA). Statistical differences between mean values were evaluated by the parametric Student *t*-test (two-tailed) where appropriate, as indicated in the figure legends, or by applying a one-way ANOVA with a Tukey post-test. Differences between the control and treated groups were considered statistically significant at *p* ≤ 0.05. The IC_50_ and CC_50_ were obtained by non-linear regressions (GraphPad Prism 8).

## Figures and Tables

**Table 1:** TPCK effect on peritoneal macrophages, *L. amazonensis* and *L. infantum* promastigotes and amastigotes, and the selectivity index.

IC_50_ ^a^/CC_50_ ^b^ (µM)				
	Promastigotes		Intracellular Amastigotes	
*L. amazonensis* (IC_50_)	PH8	Josefa	PH8	Josefa
	14.6 (10.9–19.6)	31.7 (26.8–37.6)	14.2 (7.7–26.2)	16.6 (7.9–35.2)
*L. infantum* (IC_50_)	11.3 (8.3–15.5)		21.7 (15.3–30.6)	
Peritoneal macrophages (CC_50_)	138.8 (128.4–150.1)			
**Selectivity Index (SI) ^c^**				
*L. amazonensis*	PH8	Josefa	PH8	Josefa
	9.5	4.4	9.8	8.3
*L. infantum*	12.2		6.4	

^a^ Inhibitory concentration of 50% of parasite growth (IC_50_). ^b^ Cytotoxic concentration of 50% of macrophages (CC_50_). ^c^ Selectivity index, calculated by: CC_50_ against macrophages/IC_50_ against parasite.

## Data Availability

The data presented in this study are available on request from the corresponding authors.

## Supplementary Materials

The following supporting information can be downloaded at: https://www.mdpi.com/article/10.3390/pharmaceutics14071373/s1, Figure S1: TPCK effects on AST, ALT, and creatinine levels in animals infected with *L. amazonensis*. Creatinine (A), AST (B), and ALT (C) levels were determined in the serum of animals infected with *L. amazonensis* treated with TPCK (45 and 60 mg/kg) and compared with levels in the serum of animals that did not receive the treatment. At the end of the treatment and before euthanasia, blood was collected by cardiac puncture, and the serum was separated by centrifuging the whole blood at 5000× *g* rpm for 5 min. AST, ALT, and creatinine levels were determined using kinetic detection kits obtained from Bioclin. Figure S2: TPCK effects on AST, ALT, and creatinine levels in animals infected with *L. infantum*. Creatinine (A), AST (B), and ALT (C) levels were determined in the serum of animals infected with *L. infantum* treated with TPCK (25 mg/kg) and compared to levels in the serum of animals that did not receive the treatment. At the end of the treatment and prior to euthanasia, blood was collected by cardiac puncture, and the serum was separated by centrifuging the whole blood at 5000× *g* rpm for 5 min. AST, ALT, and creatinine levels were determined using kinetic detection kits obtained from Bioclin.
