# Supplementary material for: Effects of a Serine Protease Inhibitor N-p-Tosyl-L-phenylalanine Chloromethyl Ketone (TPCK) on Leishmania amazonensis and Leishmania infantum
Source: Pharmaceutics. 2022 Jun 29;14(7):1373. doi: 10.3390/pharmaceutics14071373 (PMC9320531; doi:10.3390/pharmaceutics14071373)
Supplement: Supplementary file 1 [file pharmaceutics-14-01373-s001.zip › pharmaceutics-1684906-supplementary.pdf]

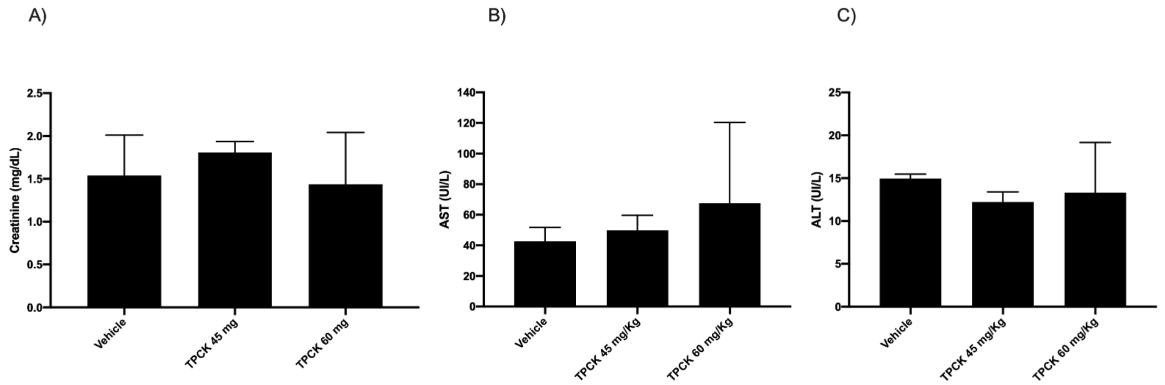

Figure S1. TPCK effects on AST, ALT, and creatinine levels in animals infected with *L. amazonensis*. Creatinine (**A**), AST (**B**), and ALT (**C**) levels were determined in the serum of animals infected with *L. amazonensis* treated with TPCK (45 and 60 mg/kg) and compared with levels in the serum of animals that did not receive the treatment. At the end of the treatment and before euthanasia, blood was collected by cardiac puncture, and the serum was separated by centrifuging the whole blood at 5000 rpm for 5 min. AST, ALT, and creatinine levels were determined using kinetic detection kits obtained from Bioclin.

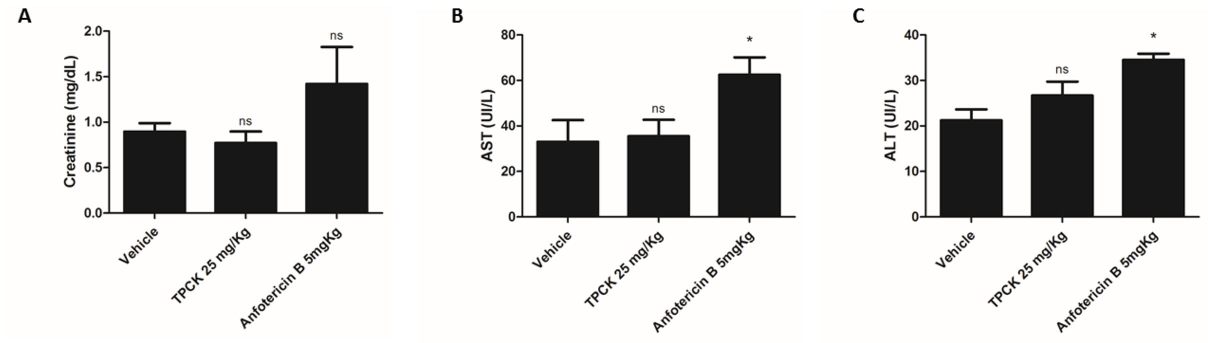

Figure S2. TPCK effects on AST, ALT, and creatinine levels in animals infected with *L. infantum*. Creatinine (**A**), AST (**B**), and ALT (**C**) levels were determined in the serum of animals infected with *L. infantum* treated with TPCK (25 mg/kg) and compared to levels in the serum of animals that did not receive the treatment. At the end of the treatment and prior to euthanasia, blood was collected by cardiac puncture, and the serum was separated by centrifuging the whole blood at 5000 rpm for 5 min. AST, ALT, and creatinine levels were determined using kinetic detection kits obtained from Bioclin.
